# Chrysotoxine Attenuates Key Atherogenic Processes via Antioxidant, Anti-Inflammatory, and COX-Dependent Antiplatelet Mechanisms

**DOI:** 10.3390/biom16030379

**Published:** 2026-03-03

**Authors:** Fozia Rustamani, Hla Nu Swe, Su Wutyi Thant, Jeeradej Moonrut, Boonchoo Sritularak, Ponlapat Rojnuckarin, Nonthaneth Nalinratana, Rataya Luechapudiporn

**Affiliations:** 1 Pharmaceutical Sciences and Technology Program, Faculty of Pharmaceutical Sciences, Chulalongkorn University, Bangkok 10330, Thailand; right2fozia@gmail.com (F.R.); hlanu.ph@gmail.com (H.N.S.); suwutyithantt@gmail.com (S.W.T.); 2 Interdisciplinary Program in Pharmacology, Graduate School, Chulalongkorn University, Bangkok 10330, Thailand; jeeradejmoonrut86@gmail.com; 3 Department of Pharmacognosy and Pharmaceutical Botany, Faculty of Pharmaceutical Sciences, Chulalongkorn University, Bangkok 10330, Thailand; boonchoo.sr@chula.ac.th; 4 Center of Excellence in Natural Products for Ageing and Chronic Diseases, Faculty of Pharmaceutical Sciences, Chulalongkorn University, Bangkok 10330, Thailand; nonthaneth.n@pharm.chula.ac.th; 5 Department of Medicine, Faculty of Medicine, Chulalongkorn University, Bangkok 10330, Thailand; rojnuckarinp@gmail.com; 6 Department of Pharmacology and Physiology, Faculty of Pharmaceutical Sciences, Chulalongkorn University, Bangkok 10330, Thailand; 7 Faculty of Pharmaceutical Sciences, Kasetsart University, Bangkok 10900, Thailand

**Keywords:** chrysotoxine, atherosclerosis, LDL oxidation, vascular inflammation, platelet activation, cyclooxygenase (COX)

## Abstract

Atherosclerosis is a complex vascular disorder driven by oxidative stress, inflammation, and platelet activation. Agents capable of targeting multiple atherogenic pathways may provide improved therapeutic benefits. In this study, we evaluated the anti-atherogenic effects of chrysotoxine, a bibenzyl compound isolated from *Dendrobium pulchellum*, using in vitro models relevant to atherogenesis. Chrysotoxine significantly suppressed hemin-induced LDL oxidation by reducing lipid peroxidation and apolipoprotein modification. In an endothelial–monocyte co-culture model, chrysotoxine markedly attenuated lipopolysaccharide-induced monocyte adhesion, indicating inhibition of endothelial inflammatory activation. Chrysotoxine also inhibited platelet aggregation induced by arachidonic acid, ADP, and collagen in a concentration-dependent manner, with the strongest effects observed against arachidonic acid–mediated responses, suggesting modulation of the thromboxane pathway. Molecular docking analyses and cyclooxygenase activity assays further indicated that chrysotoxine may interact with both COX-1 and COX-2, exhibiting inhibitory activity in the low micromolar range. Collectively, these findings demonstrate that chrysotoxine modulates multiple key processes involved in atherogenesis, including oxidative LDL modification, vascular inflammation, and platelet activation. Although further in vivo studies are required, chrysotoxine represents a promising plant-derived candidate for the development of multi-target strategies against atherosclerotic disease.

## 1. Introduction

Atherosclerosis is a chronic inflammatory disease of the arterial wall that develops progressively in medium- and large-sized arteries, leading to the accumulation of lipid-rich and fibrotic plaques [1,2]. It is the major underlying cause of cardiovascular diseases and a leading contributor to global mortality [3]. The disease is initiated by modifications of LDL within the arterial wall [4], which trigger local inflammation and increase the release of chemotactic mediators and adhesion molecules on endothelial cells [5]. These changes promote the recruitment of circulating monocytes to developing lesions during atherogenesis [6,7,8]. Platelets are now recognized as key contributors to atherogenesis, as elevated levels of activated platelets in the circulation can accelerate plaque growth and disease progression. Under normal conditions, platelets help maintain vascular integrity by preventing blood loss; however, when endothelial injury or plaque rupture exposes subendothelial collagen, platelets become activated and aggregate to form thrombi [9]. Excessive platelet activation, in turn, contributes to cardiovascular complications such as thrombosis, ischemic stroke, myocardial infarction, and the progression of atherosclerosis. Cyclooxygenase (COX) enzymes are key regulators of inflammation, platelet function, and vascular homeostasis, making them important therapeutic targets in cardiovascular disease [10,11]. While COX-1 inhibition by aspirin is widely exploited to reduce thrombotic risk, selective COX-2 inhibition has been associated with adverse cardiovascular outcomes, primarily due to diminished endothelial prostacyclin production and the consequent loss of its vasoprotective effects. In contrast, non-selective COX inhibition may attenuate inflammatory prostanoid signaling and confer antithrombotic benefits; however, this approach is also associated with an increased risk of NSAID-related adverse effects, particularly gastrointestinal toxicity linked to COX-1 inhibition [12,13,14,15,16,17]. Therefore, the non-selective COX inhibition observed in this study warrants careful in vivo evaluation to balance therapeutic efficacy with safety.

Natural products are a valuable source of bioactive molecules for drug discovery, particularly for developing new therapies for cardiovascular diseases [18]. Therapies based on natural compounds have shown clear benefits in the management of cardiovascular diseases [19]. *Dendrobium*, one of the largest genera in the Orchidaceae family, has a long history of use in traditional Chinese medicine [20]. Ethnopharmacological studies have reported its use in treating various conditions, including cardiovascular diseases [20,21,22,23,24,25].

**Figure 1:**
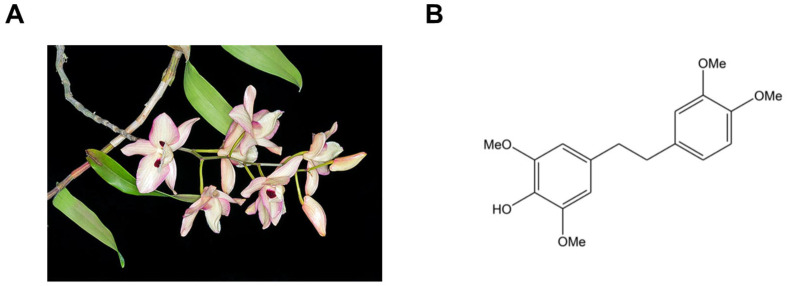
Source and chemical structure of chrysotoxine. (**A**) *Dendrobium pulchellum*, the plant species from which chrysotoxine is naturally derived. (**B**) The chemical structure of chrysotoxine.

Among the bioactive constituents of *Dendrobium* species, bibenzyl compounds have gained attention for their unique structures and diverse biological activities. These phenolic compounds exhibit antioxidant [26], anti-inflammatory [27], antibacterial [27], antidiabetic [28], and anticancer activities [29]. One bibenzyl compound of particular interest is Chrysotoxine (4-[2-(3,4-dimethoxyphenyl)ethyl]-2,6-dimethoxyphenol), a well-characterized molecule isolated from *Dendrobium pulchellum* (Figure 1) and reported to display strong biological activity in in vitro cell-based studies [30,31,32]. Despite its promising pharmacological profile, the involvement of chrysotoxine in atherogenesis has not yet been explored. While its antioxidant properties have been described, its effects on platelet function, LDL oxidation, and other atherosclerosis-related processes have not been systematically investigated. The present study was designed to address these gaps.

In this study, we investigated the ability of chrysotoxine to modulate key processes involved in atherogenesis using in vitro models. Its effects on LDL oxidation were assessed in hemin-induced ox-LDL using the TBARS assay and relative electrophoretic mobility analysis. Hemin was selected as an oxidative inducer because it represents a redox-active heme moiety released from hemoproteins during hemolysis or intraplaque hemorrhage, condition commonly associated with advanced atherosclerotic lesions [33]. The protective effects of chrysotoxine on monocyte endothelial cell adhesion were investigated in LPS-induced endothelial cells. Platelet activation was evaluated using the Born turbidometric light transmission aggregometry assay to assess inhibition of agonist-induced platelet aggregation. Interactions with COX-1 and COX-2 were explored through in silico molecular docking, focusing on binding affinity, binding poses, and key interactions, and were further supported by cyclooxygenase inhibition assays. Together, these results identify chrysotoxine as a plant-derived compound with multifaceted anti-atherogenic and protective properties, highlighting its potential as a promising therapeutic candidate. To our knowledge, this is the first study to comprehensively evaluate the effects of chrysotoxine on oxidative LDL modification, endothelial inflammation, platelet function, and COX activity in the context of atherogenesis.

## 2. Materials and Methods

### 2.1. Chemicals

All chemicals and reagents employed in this study were of analytical grade and freshly prepared according to the manufacturers’ instructions. Detailed information on the reagents, including supplier names and catalog numbers, is provided in Supplementary Table S1.

### 2.2. Isolation of Chrysotoxine

Chrysotoxine (CTX) (molecular weight: 318.4 g/mol) was isolated from *Dendrobium pulchellum* following a previously reported procedure [34]. The dried stems of *Dendrobium pulchellum* (0.5 kg) were finely powdered and extracted at room temperature with 95% ethanol. The extraction was carried out three times, each using 10 L of solvent. After filtration, the combined ethanolic extracts were concentrated under reduced pressure to yield a crude residue (50 g). The crude extract was fractionated by vacuum liquid chromatography (VLC) on silica gel using a gradient system of n-hexane–ethyl acetate, followed by methanol, to obtain seven fractions (A–G). Fraction D (1.8 g) was selected for further purification and subjected to gel filtration over Sephadex LH-20, eluted with a methanol–dichloromethane mixture (1:1, *v*/*v*), resulting in eight subfractions (D-1 to D-8). Subfraction D-4 (330 mg) was further separated by column chromatography on silica gel using n-hexane–ethyl acetate (7:3, *v*/*v*) as the eluent, affording several fractions. Combined fractions 6–9 (35 mg) were subsequently purified by repeated silica gel column chromatography under the same solvent conditions. This final purification step yielded chrysotoxine (11 mg) as a pure compound. The structure of chrysotoxine was confirmed by spectroscopic analyses, including NMR and mass spectrometry.

### 2.3. Subjects

The study recruited healthy volunteers aged 18 to 50 years who had no known medical conditions. Participants were non-smokers, did not consume alcohol and had avoided taking any medication for at least two weeks before the experiment. All individuals provided written informed consent prior to participation. The research protocol was reviewed and approved by the Institutional Review Board, Faculty of Medicine, Chulalongkorn University (COA No. 1084/2024, approved on 8 August 2024; COA No. 493/2020, approved on 20 April 2020).

### 2.4. Preparation of Low Density Lipoprotein (LDL)

The experiments were conducted using human low-density lipoprotein (LDL) isolated from healthy volunteers, as described in Section 2.3. LDL was obtained from pooled plasma collected from overnight-fasting individuals. It is well documented that young women generally exhibit lower LDL and higher HDL levels compared with young men [35]. Female donors were selected to reduce inter-experimental variability; however, LDL lipid composition is not expected to differ significantly between sexes under fasting conditions. Hormonal status was not controlled in this study and could contribute to inter-donor variability.

Briefly, 30 mL of blood from each donor was collected into tubes containing Na_2_EDTA as an anticoagulant (final concentration: 1 mg/mL blood). Plasma was separated by centrifugation at 2454× *g* for 15 min at 4 °C (Falcon: 6300) and stored at −80 °C until further processing. LDL was subsequently isolated by sequential density gradient ultracentrifugation using a Hitachi CP100 NX ultracentrifuge (Hitachi High-Tech Corporation, Tokyo, Japan) equipped with a P100AT2 fixed-angle rotor (Himac, Tokyo, Japan). Plasma density was adjusted to 1.019–1.063 g/mL using KBr solutions (30.6 and 90.3 g/L, respectively) containing 0.05% (*w*/*v*) EDTA (pH 7.0) prepared in ddH_2_O, and plasma was diluted in 0.154 M NaCl. Ultracentrifugation was performed at 289,000× *g* at 16 °C to isolate the LDL fraction [36].

The isolated LDL was dialyzed overnight against 10 mM phosphate-buffered saline (PBS, pH 7.4) to remove residual EDTA and salts that could interfere with subsequent oxidation, thereby ensuring controlled and reproducible oxidation conditions. LDL protein concentration was determined using a NanoDrop UV–visible spectrophotometer (Thermo Fisher Scientific, Waltham, MA, USA) [37].

### 2.5. Hemin Induced Low Density Lipoprotein (LDL) Oxidation

For oxidation experiments, LDL was adjusted to a final concentration of 400 µg/mL and pre-incubated with different concentrations of CTX for 30 min at 37 °C in a shaking incubator. Oxidative modification of LDL was initiated by the addition of hemin (5 µM) and allowed to proceed at 37 °C for up to 24 h. Trolox (1 µM) was included as a positive antioxidant control. At predetermined time points (0, 1, 3, 6, 12, and 24 h), aliquots were withdrawn, and the oxidation reaction was terminated by the addition of EDTA (100 µM) and butylated hydroxytoluene (BHT, 5 mM) to chelate metal ions and prevent further oxidative reactions. Oxidized LDL samples were subsequently used for biochemical analyses, including TBARS measurement.

### 2.6. Determination of Lipid Peroxidation by TBARs Assay

This method is based on the principle that malondialdehyde (MDA), a by-product of lipid peroxidation, reacts with thiobarbituric acid (TBA) and produces thiobarbituric acid-reactive substance (TBARS), a pink-colored adduct. Although TBARS is a non-specific marker of lipid peroxidation, it remains a widely accepted method for monitoring oxidative LDL modification.

100 mM butylated hydroxytoluene (BHT), 10% trichloroacetic acid (TCA), 5 mM ethylene diamine tetra acetic acid (EDTA), 8% sodium dodecyl sulphate (SDS) and 6% thiobarbituric acid (TBA) were added and mixed well. Then the mixture was heated at 95 °C for 1 h. After that, this mixture was cooled for 10 min and then added butan-1-ol and centrifuged. TBARs formation was measured by spectrofluorometer, excitation and emission wavelength at 515 nm and 553 nm, respectively [38].

TBARS levels were normalized to LDL protein content and expressed as nanomoles of TBARS per milligram LDL protein. All measurements were conducted within the linear range of the assay using Tetramethoxypropane (TMP) as a standard to generate a calibration curve. Reagent blanks and appropriate controls were included to correct for background fluorescence. In addition, butylated hydroxytoluene (BHT) and EDTA were included in the reaction mixture to minimize artifactual lipid peroxidation during sample processing.

### 2.7. Relative Electrophoretic Mobility (REM) of LDL

Agarose gel electrophoresis was used to determine the negative charge modification of LDL and it is used as indicator of protein oxidation. Oxidative modification of apolipoprotein in LDL involves derivatization and loss of positively charged lysine residues, which increases the net negative charge of LDL particles and leads to faster migration during agarose gel electrophoresis compared with native LDL [39].

Native LDL and hemin-induced ox-LDL (he-oxLDL) collected at different incubation times were separated on a 1% agarose gel prepared in Tris-acetate-EDTA (TAE) buffer. Electrophoresis was performed at a constant voltage of 70 V for 45 min. After separation, LDL bands were visualized by staining with Coomassie Brilliant Blue for 30 min, followed by destaining with water until minimal background was observed [37].

The migration distance of each LDL sample from the origin was measured. Relative electrophoretic mobility (REM) was calculated as the ratio of the migration distance of oxidized LDL to that of native LDL run on the same gel, with native LDL used as the reference (REM = 1.0). Increased REM values were interpreted as increased negative charge modification of LDL.

### 2.8. Cell Culture

The human endothelial cell line EA.hy926 (ATCC^®^ CRL-2922™, Manassas, VA, USA) was cultured in Dulbecco’s Modified Eagle Medium (DMEM) supplemented with 10% heat-inactivated fetal bovine serum (FBS) and 1% penicillin–streptomycin. Cells were maintained at 37 °C in a humidified incubator with 5% CO_2_. For routine subculture, cells were detached using trypsin–EDTA and seeded at an appropriate density. The human monocytic cell line THP-1 (ATCC^®^ TIB-202™, Manassas, VA, USA) was used as a model for circulating monocytes in the adhesion assay. THP-1 cells were maintained in RPMI-1640 medium containing 10% FBS and 1% penicillin–streptomycin under standard culture conditions (37 °C, 5% CO_2_). As THP-1 cells grow in suspension, gentle centrifugation was used during passaging, and fresh medium was supplied every 2–3 days to maintain cell viability.

### 2.9. Cell Viability Assay

Cell viability was assessed using the MTT assay. Cells were first seeded in 96-well plates and allowed to attach overnight in complete growth medium. After 24 h, the culture medium was replaced with medium containing 1% serum, and the cells were exposed to either 0.5% DMSO as the vehicle control or varying concentrations of chrysotoxine for an additional 24 h. After treatment, MTT reagent (0.5 mg/mL in PBS) was added to each well, followed by incubation at 37 °C for 2 h. The formed formazan crystals were then dissolved in DMSO, and absorbance was recorded at 570 nm using a microplate reader. Cell viability was expressed as a percentage relative to the untreated control group.

### 2.10. Monocyte Adhesion Assay

Monocyte adhesion to EA.hy926 endothelial cells was assessed using a previously described method [40,41]. EA.hy926 cells were seeded at a density of 1 × 10^5^ cells per well in 24-well plates and allowed to adhere overnight at 37 °C in a humidified atmosphere containing 5% CO_2_. The following day, cells were pre-treated with chrysotoxine at concentrations of 1, 5, and 10 µM for 30 min, followed by stimulation with LPS (1 µg/mL) for 24 h in the continued presence of chrysotoxine (co-treatment).

THP-1 monocytes were harvested at a density of 2 × 10^5^ cells per well, centrifuged at 200× *g* for 5 min, and resuspended in 1 mL RPMI medium. Cells were labeled with Calcein-AM (5 µM) for 30 min in the dark, followed by replacement with fresh RPMI medium and incubation for an additional 30 min to remove excess AM ester. Labeled THP-1 cells were centrifuged again at 200× *g* for 5 min and resuspended in 12 mL RPMI medium. Labeled monocytes were then added to the pre-treated EA.hy926 cells and co-cultured for 1 h at 37 °C. Non-adherent cells were removed by gentle washing with PBS, and the number of adherent monocytes was quantified using a fluorescence microscope. Dexamethasone (10 µM) was used as positive control.

### 2.11. Blood Collection and Platelet-Rich Plasma (PRP) Preparation

For each participant, approximately 30 mL of venous blood was drawn into plastic tubes containing 3.2% sodium citrate, maintaining a 9:1 ratio of blood to anticoagulant (*v*/*v*). Platelet-rich plasma (PRP) was obtained by centrifuging the blood at 200× *g* for 10 min at 21 °C, after which the upper plasma fraction was carefully collected. The remaining sample was further centrifuged at 1500× *g* for 15 min at 21 °C to obtain platelet-poor plasma (PPP), which was used as the reference for 100% light transmission. Platelet aggregation tests were performed no sooner than 15 min after PRP preparation to allow recovery from transient refractoriness, and all assays were completed within three hours of blood collection.

### 2.12. Platelet Aggregation Assay

Platelet aggregation was assessed following a previously established procedure [42], using the Born turbidimetric method with an AggRAM™ aggregometer (Helena Laboratories, Beaumont, TX, USA). 200 µL of platelet-rich plasma (PRP) served as the baseline for 0% light transmission, while 250 µL of platelet-poor plasma (PPP) was used to define 100% transmission. Light transmission at 600 nm was continuously recorded for six minutes after adding 25 µL of the selected agonist. A submaximal concentration of agonists (ADP 4 µM, collagen 2 µg/mL, and arachidonic acid 0.5 mM) were used to stimulate platelet aggregation. Prior to agonist addition, PRP samples were incubated with 25 µL of chrysotoxine for five minutes at 37 °C. A vehicle control containing 0.5% DMSO and a positive control with 0.1 mM aspirin were included for comparison. The extent of platelet aggregation was determined based on the increase in light transmission and expressed as a percentage of maximal aggregation.

### 2.13. Molecular Docking

To explore how chrysotoxine interacts with the active sites of COX-1 and COX-2, molecular docking studies were carried out using AutoDock Vina (AutoDockTools 1.5.6., Scripps research, San Diego, CA, USA) [43]. The crystal structures of COX-1 (PDB ID: 2OYE) and COX-2 (PDB ID: 1CX2) were retrieved from the Protein Data Bank and pre-processed using the Protein Preparation Wizard to remove water molecules, add hydrogens, and optimize the structure. The 3D structure of chrysotoxine was obtained from the PubChem database and prepared for docking following standard protocols. Docking simulations were then performed to estimate the binding affinities, where more negative values corresponded to stronger ligand–enzyme interactions. The resulting docking poses and molecular interactions between chrysotoxine and the catalytic residues of COX-1 and COX-2 were examined and visualized using Discovery Studio 2017 (Accelrys, Inc., San Diego, CA, USA) [42].

### 2.14. Cyclooxygenase Activity Assay

Chrysotoxine was preincubated with either COX-1 or COX-2 enzymes for 5 min before initiating the reaction. Arachidonic acid and 10-acetyl-3,7-dihydroxyphenoxazine (ADHP) were then added and allowed to react for 2 min. During this process, prostaglandin G_2_ reacts with ADHP to generate resorufin, a fluorescent compound. The fluorescence intensity of resorufin was recorded at an excitation wavelength of 530–540 nm and an emission wavelength of 585–595 nm. The assay was carried out using a COX fluorescent inhibitor screening kit (Cayman Chemical, Ann Arbor, MI, USA) according to the manufacturer’s instructions.

### 2.15. Statistical Analysis

All results are presented as the mean values with their corresponding standard errors of the mean (SEM). Statistical comparisons among groups were performed using one-way analysis of variance (ANOVA), followed by Dunnett’s post hoc test. A *p*-value below 0.05 was taken to indicate statistical significance.

## 3. Results

### 3.1. Protective Effect of Chrysotoxine on Hemin-Induced Low-Density Lipoprotein (LDL) Oxidation

#### 3.1.1. Effect of Chrysotoxine on TBARs Formation

**Figure 2:**
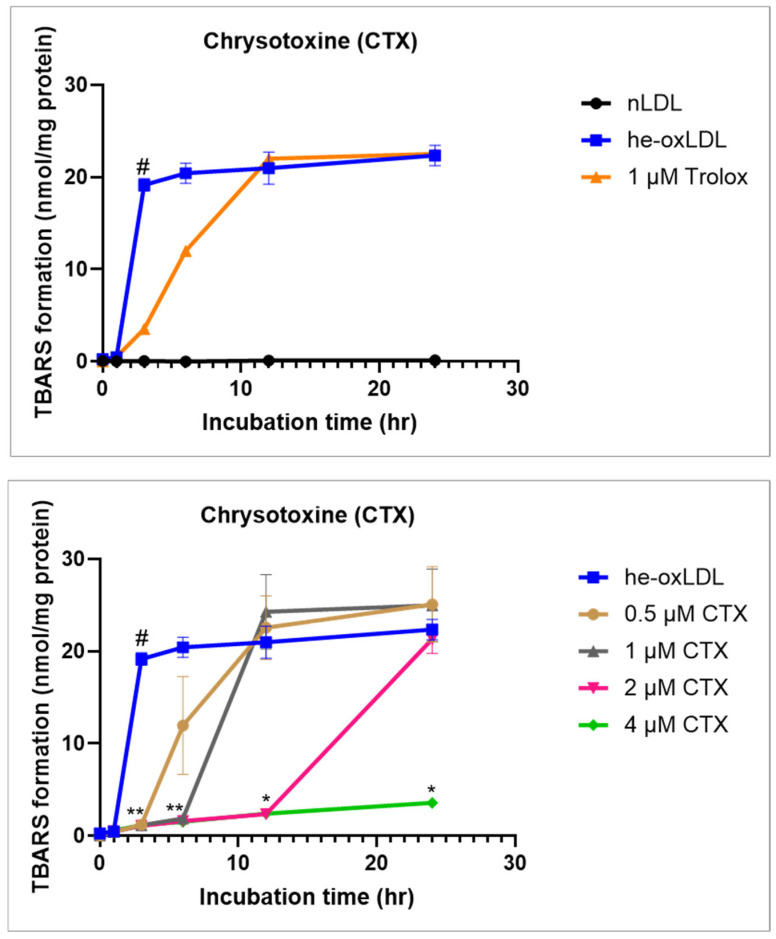
Effects of chrysotoxine on TBARS formation in hemin-induced ox-LDL. LDL samples were pretreated with chrysotoxine or Trolox (positive control) for 30 min prior to the addition of hemin to initiate oxidative modification. TBARs levels were subsequently measured at multiple time points over a 24 h incubation period using a TBARS assay. Results are expressed as mean ± SEM (n = 3). # *p* < 0.05 compared with native LDL (nLDL); * *p* < 0.05 and ** *p* < 0.01 compared with hemin-induced ox-LDL.

The formation of thiobarbituric acid–reactive substances (TBARS) during hemin induced LDL oxidation is shown in Figure 2. Exposure of LDL to hemin resulted in a rapid depletion of endogenous α-tocopherol, followed by a pronounced increase in lipid peroxidation. TBARS levels were therefore monitored at multiple time points over a 24 h incubation period to characterize the temporal profile of LDL oxidation.

In hemin-induced ox-LDL, TBARS concentrations increased sharply during the early phase of oxidation, rising from 0.27 ± 0.10 nmol/mg protein at 0 h to 0.46 ± 0.11 nmol/mg protein at 1 h, and reaching 18.92 ± 4.29 nmol/mg protein by 3 h. TBARS accumulation reached a maximal level by 6 h (20.45 ± 4.56 nmol/mg protein) and remained relatively stable thereafter, indicating an early plateau in lipid peroxidation under these experimental conditions.

Chrysotoxine (CTX) pretreatment (30 min) reduced TBARS formation during hemin-induced LDL oxidation in a concentration- and time-dependent manner. Relative to hemin-induced ox-LDL, CTX at 2 and 4 µM attenuated TBARS accumulation at early-to-intermediate time points (notably 3–6 h). At 2 µM, TBARS remained lower during the early phase but gradually increased at later time points (12–24 h), whereas 4 µM produced a more sustained suppression across the full 24 h incubation period. The delayed increase at the lower concentration likely reflects incomplete suppression of cumulative lipid peroxidation under continued hemin-driven oxidative conditions when CTX is present at a submaximal level. Overall, these data indicate a concentration-dependent inhibitory effect of CTX on hemin-induced LDL lipid peroxidation.

Trolox also significantly reduced TBARS formation during the early phase of LDL oxidation, with a clear inhibitory effect observed up to 6 h. However, this effect was no longer detectable at 12 or 24 h, indicating limited persistence of antioxidant protection under prolonged oxidative conditions.

To exclude the possibility of incubation-related artifacts, native LDL was incubated in parallel under identical conditions in the absence of hemin. TBARS levels in nLDL remained low and unchanged throughout the incubation period, whereas hemin-induced ox-LDL showed a clear time-dependent increase in TBARS formation. These findings confirm that the observed lipid peroxidation resulted from hemin-induced oxidative modification of LDL rather than prolonged incubation or assay-related interference.

#### 3.1.2. Effect of Chrysotoxine on Relative Electrophoretic Mobility (REM)

**Figure 3:**
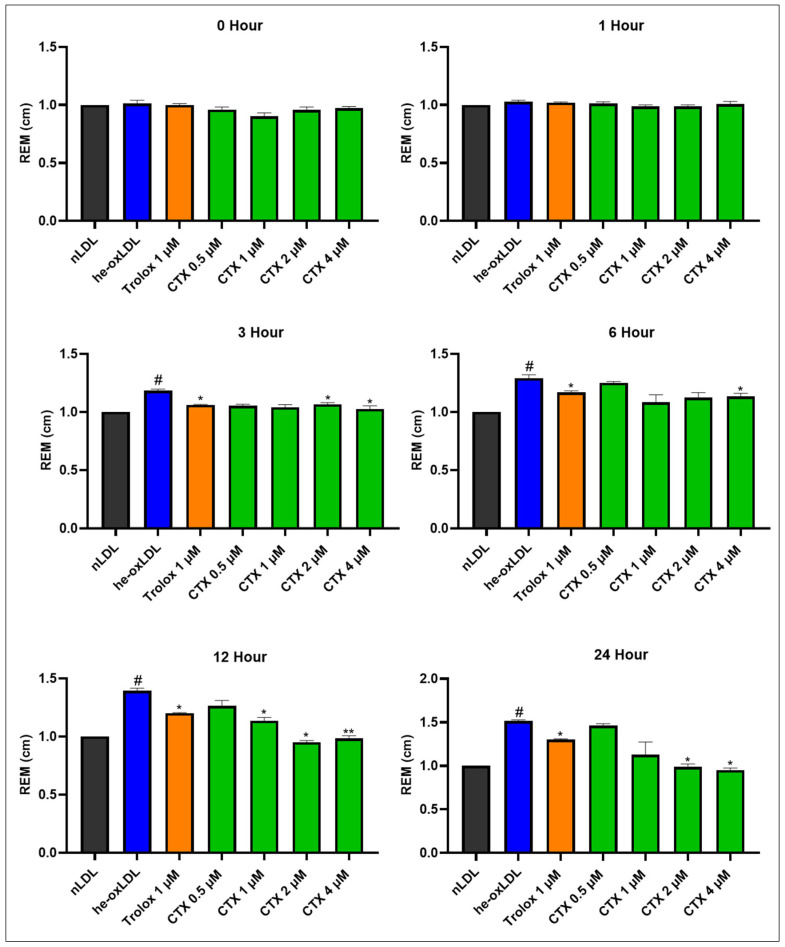
Effect of chrysotoxine on REM in hemin-induced LDL oxidation. REM was assessed at different incubation times and expressed as mean ± SEM (n = 3). # *p* < 0.05 vs. native LDL (nLDL); * *p* < 0.05, ** *p* < 0.01 vs. hemin-induced ox-LDL (he-oxLDL). REM, an indicator of apolipoprotein oxidation, was determined by agarose gel electrophoresis. Relative electrophoretic mobility (REM) was calculated as REM = (migration distance of sample LDL band)/(migration distance of native LDL band) measured on the same gel.

Oxidative modification of apolipoproteins in LDL was assessed by changes in relative electrophoretic mobility (REM), as shown in Figure 3. REM was assessed at different incubation times (Supplementary Figure S1). Hemin treatment caused a time-dependent increase in REM, with values rising to 1.18 ± 0.01, 1.30 ± 0.07, 1.33 ± 0.06, and 1.51 ± 0.01 at 3, 6, 12, and 24 h, respectively. These findings demonstrate that hemin progressively promotes protein oxidation in LDL. Trolox at 1 µM effectively attenuated this oxidative modification, with the protective effect maintained for 24 h. Chrysotoxine also reduced protein oxidation in a concentration-dependent manner, with higher concentrations (2 and 4 µM) significantly lowering REM values. The duration of protection provided by chrysotoxine was comparable to that observed with Trolox.

### 3.2. Protective Effects of Chrysotoxine on LPS-Stimulated Endothelial Inflammation

#### 3.2.1. Effect of Chrysotoxine on Cell Viability

**Figure 4:**
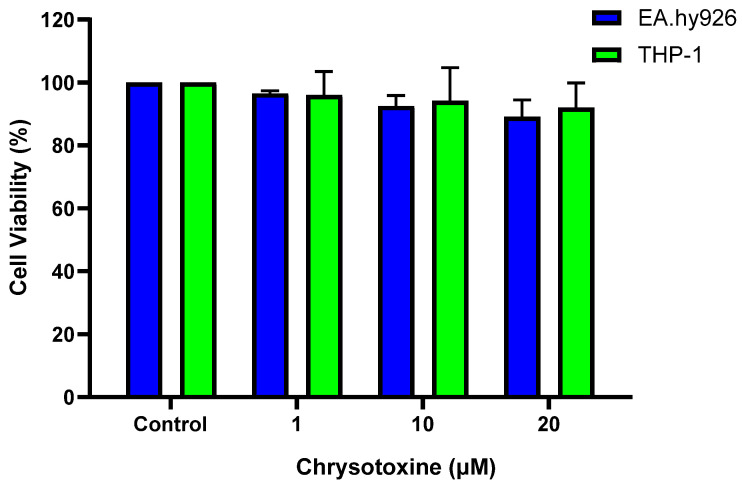
Effects of chrysotoxine on cell viability. EA.hy926 endothelial cells and THP-1 monocytes were seeded and treated with various concentrations of chrysotoxine for 24 h. Cell viability is expressed as a percentage of the control and presented as mean ± SEM (n = 3).

The effect of chrysotoxine on cell viability of EA.hy926 and THP-1 cells were determined (Supplementary Figure S2). The cells were seeded and treated for 24 h treatment with various concentrations of the chrysotoxine and the cell viability was assessed using MTT assay. As illustrated in Figure 4, chrysotoxine did not cause any noticeable reduction in cell viability under these experimental conditions.

#### 3.2.2. Effect of Chrysotoxine on Monocyte Adhesion

**Figure 5:**
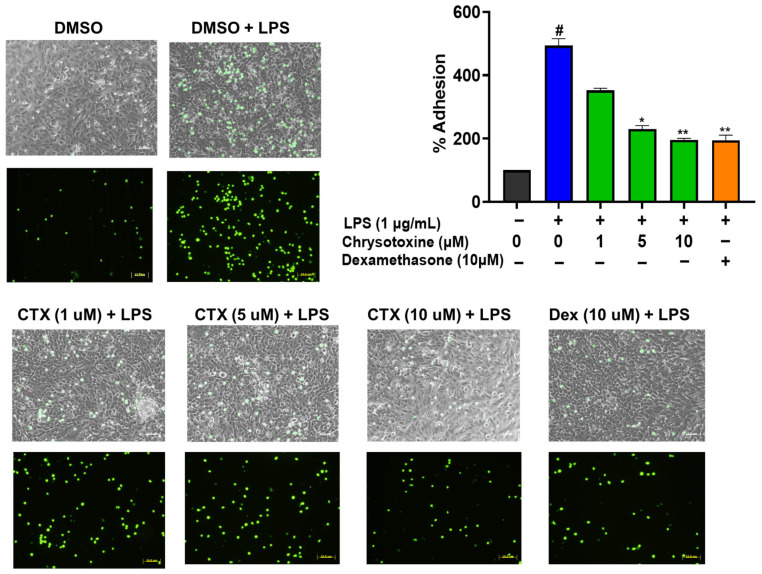
Effects of chrysotoxine on monocyte adhesion to LPS-stimulated endothelial cells. EA.hy926 endothelial cells were pretreated with chrysotoxine (1, 5, and 10 µM) and then stimulated with LPS (1 µg/mL) for 24 h. Dexamethasone (10 µM) was used as a positive control. Calcein AM–labeled THP-1 cells were subsequently added for 1 h, and monocyte adhesion was evaluated by fluorescence microscopy (10× magnification). Results are expressed as mean ± SEM (n = 3). # *p* < 0.01 vs. control; * *p* < 0.05, ** *p* < 0.01 vs. LPS-treated group. Scale bar = 10.0 μm.

To evaluate the anti-inflammatory activity of chrysotoxine, its effect on monocyte adhesion to EA.hy926 endothelial cells stimulated with LPS was examined. As expected, exposure to LPS significantly increased monocyte adhesion, confirming endothelial activation. Pre-treatment with chrysotoxine significantly reduced monocyte attachment in a concentration-dependent manner. Notably, treatment with 5 µM and 10 µM chrysotoxine markedly lowered monocyte adhesion compared with LPS treatment alone (Figure 5). These findings indicate that chrysotoxine can suppress endothelial inflammatory responses by inhibiting monocyte adhesion to activated endothelial cells. These effects may involve suppression of endothelial adhesion molecule expression, although this was not directly assessed.

### 3.3. Protective Effects of Chrysotoxine on Platelet Activation

#### Effects of Chrysotoxine on Agonist-Induced Platelet Aggregation

**Figure 6:**
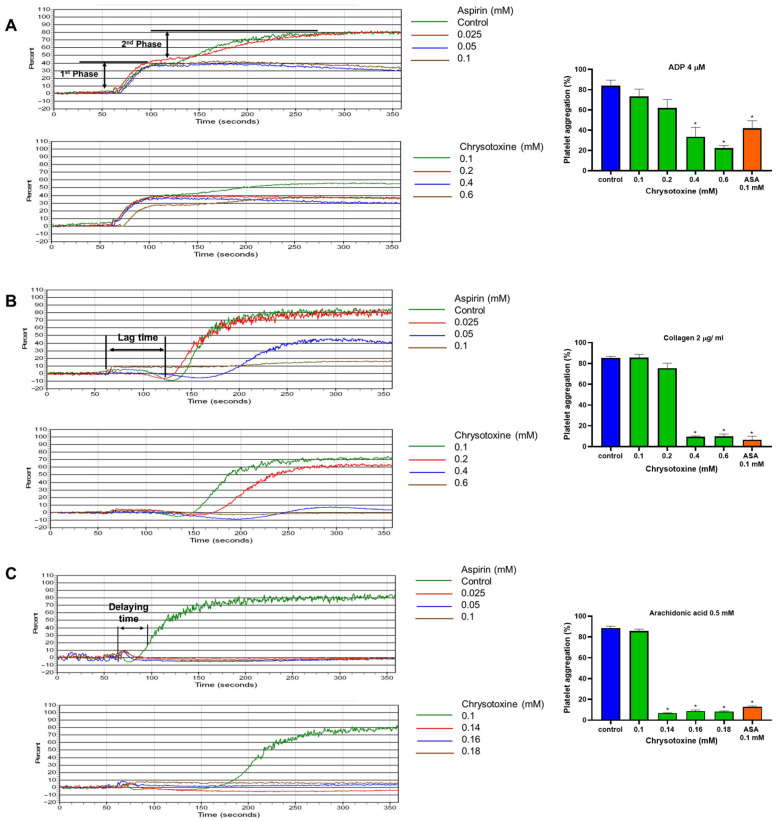
Effects of chrysotoxine on platelet aggregation induced by different agonists. Platelets were preincubated with chrysotoxine, aspirin (positive control), or 0.5% DMSO (vehicle control) at 37 °C for 5 min before stimulation. Aggregation was then triggered by (**A**) ADP (4 µM), (**B**) collagen (2 µg/mL), or (**C**) arachidonic acid (0.5 mM). Results are expressed as percentage aggregation (mean ± SEM, n = 3). * *p* < 0.05 versus vehicle control.

As illustrated in Figure 6A, Chrysotoxine preferentially attenuated the secondary amplification phase of ADP-induced platelet aggregation, while exerting a comparatively weaker effect on the initial phase. In the case of arachidonic acid-stimulated aggregation, chrysotoxine markedly prolonged the lag time relative to the control group (Figure 6B). Similarly, chrysotoxine at a concentration of 0.4 mM and 0.6 mM significantly delayed the onset of collagen-induced platelet aggregation compared with the vehicle-treated group (Figure 6C). Quantitative analysis revealed that chrysotoxine inhibited platelet aggregation triggered by arachidonic acid, collagen, and ADP with differing potencies, as reflected by IC_50_ values of 0.126 ± 0.001 mM, 0.27 ± 0.128 mM, and 0.41 ± 0.017 mM, respectively (Supplementary Figure S3).

### 3.4. Molecular Docking Studies of Chrysotoxine with Cyclooxygenase Enzymes

**Figure 7:**
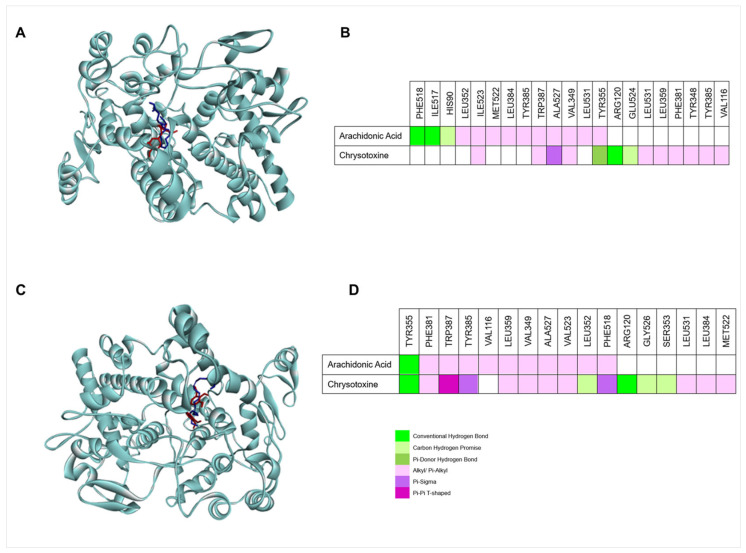
Molecular docking of chrysotoxine into the COX-I and COX-II enzymes. (**A**) Three-dimensional representation showing the binding positions of chrysotoxine (red) and arachidonic acid (blue) within the COX-1 active site. (**B**) Interacting amino acid residues of COX-1 involved in the binding of arachidonic acid and chrysotoxine. (**C**) Three-dimensional visualization of chrysotoxine (red) and arachidonic acid (blue) within the COX-2 active site. (**D**) Interacting amino acid residues of COX-2 involved in the binding of arachidonic acid and chrysotoxine.

Molecular docking is a computational approach used to examine interactions between small molecules and target proteins by predicting ligand binding and interaction strength. In this study, docking analyses were performed using AutoDock to evaluate the binding of chrysotoxine to cyclooxygenase-1 (COX-1) and cyclooxygenase-2 (COX-2) as shown in Figure 7.

#### 3.4.1. Molecular Docking of Chrysotoxine with COX-1

To validate the docking protocol, the native ligand from the COX-1 crystal structure (PDB ID: 2OYE) was re-docked into its binding site. The predicted pose closely matched the crystal conformation, with a low RMSD of 0.657 Å, confirming the reliability of the method (Supplementary Figure S4). Arachidonic acid, used as the reference ligand, was docked into the COX-1 active site and formed hydrogen bonds with PHE518 and ILE517, a C–H interaction with HIS90, and multiple hydrophobic interactions with key residues involved in substrate recognition and enzyme activity.

Chrysotoxine also bound stably within the COX-1 active site, exhibiting a binding energy of −8.1 kcal/mol. It formed a hydrogen bond with ARG120, a C–H interaction with GLU524, and a π-donor hydrogen bond with TYR355, along with a π–σ interaction with ALA527 and several hydrophobic contacts. Notably, its binding site closely overlapped with that of arachidonic acid and showed slightly stronger affinity (−8.1 vs. −7.8 kcal/mol), suggesting a stable and potentially effective interaction with COX-1.

#### 3.4.2. Molecular Docking of Chrysotoxine with COX-2

To validate the docking approach, the native ligand from the COX-2 crystal structure (PDB ID: 4COX) was re-docked into the active site, yielding a low RMSD of 0.457 Å and confirming the reliability of the protocol (Supplementary Figure S5). Docking analysis showed that chrysotoxine binds stably within the COX-2 active site with a binding energy of −7.1 kcal/mol. It formed hydrogen bonds with ARG120 and TYR355, along with C–H, aromatic, and hydrophobic interactions that stabilized the ligand in the catalytic pocket.

In comparison, arachidonic acid displayed a slightly lower binding affinity (−7.7 kcal/mol) and interacted with similar residues. The overlap in binding sites suggests that chrysotoxine occupies the same catalytic region as the natural substrate and may competitively inhibit COX-2 activity. The docking scores are method-dependent and do not directly represent experimental binding affinities, and the predictions may be affected by limited receptor flexibility, ligand (substrate) conformational flexibility, and simplified solvation/water-mediated interactions [44].

### 3.5. Effects of Chrysotoxine on Cyclooxygenase (COX) Enzymes Activities

**Figure 8:**
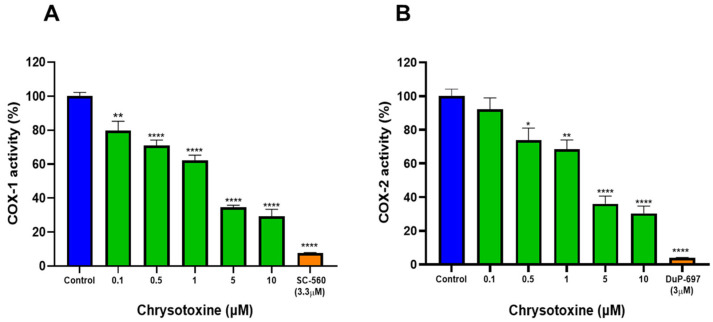
Effect of chrysotoxine on COX-1 and COX-2 enzyme activities. Chrysotoxine was pre-incubated with (**A**) COX-1 and (**B**) COX-2 in assay buffer for 5 min, after which enzyme activity was quantified using a COX fluorescent inhibitor screening assay. SC-560 and DuP-697 were included as selective reference inhibitors for COX-1 and COX-2, respectively. Data are shown as (mean ± SEM, n = 3). * *p* < 0.05, ** *p* < 0.001, **** *p* < 0.0001 versus vehicle control.

The inhibitory activity of chrysotoxine toward cyclooxygenase (COX) isoforms was assessed using a COX fluorimetric inhibitor screening assay. As shown in Figure 8, chrysotoxine suppressed both COX-1 and COX-2 activities in a concentration-dependent manner. Nonlinear regression analysis of the dose–response curves yielded IC_50_ values of 1.03 µM for COX-1 and 1.30 µM for COX-2 (Supplementary Figure S6).

The selectivity of COX inhibition was evaluated by calculating the selectivity index (SI), defined as the ratio of IC_50_ values for COX-1 to COX-2, a commonly used parameter for assessing isoform preference [45,46]. Based on the obtained IC_50_ values, the calculated COX-1/COX-2 ratio was 0.79, indicating a lack of pronounced selectivity toward either COX isoform. Assay validity and enzyme specificity were confirmed using SC-560 and DuP-697 as selective reference inhibitors for COX-1 and COX-2, respectively.

Collectively, these findings demonstrate that chrysotoxine directly inhibits both COX isoforms at low micromolar concentrations, suggesting that its antiplatelet effects may be partially mediated through modulation of COX-dependent pathways, including reduced thromboxane synthesis and platelet activation.

## 4. Discussion

Atherosclerosis is a multifactorial vascular disease driven by the interplay between oxidative stress, endothelial inflammation, and platelet activation. Therapeutic strategies that concurrently modulate these interconnected processes may therefore offer enhanced protective potential. In the present study, we investigated the effects of chrysotoxine, a bibenzyl compound isolated from *Dendrobium pulchellum*, on key mechanisms implicated in atherogenesis using complementary in vitro and computational approaches.

Oxidative modification of low-density lipoprotein (LDL) is a key initiating event in atherosclerotic plaque development [4,47,48,49]. LDL is highly susceptible to iron-dependent oxidative stress, particularly in the presence of redox-active heme species such as hemin released during hemolytic or hemorrhagic conditions [50,51,52]. Hemin can associate with LDL and catalyze Fenton-like reactions, promoting lipid peroxidation and oxidative modification of apolipoprotein B-100, leading to the formation of cytotoxic oxidized LDL (oxLDL) species implicated in foam cell formation and endothelial dysfunction [37,53,54]. In this study, hemin exposure induced oxidative modification of LDL, evidenced by a time-dependent increase in TBARS formation and enhanced relative electrophoretic mobility, reflecting lipid peroxidation and apolipoprotein alteration [33,37].

Chrysotoxine significantly attenuated TBARS formation and reduced electrophoretic mobility in a concentration-dependent manner, indicating protection against both lipid and associated protein oxidation. The sustained inhibitory effect during prolonged incubation suggests interference with the propagation phase of lipid peroxidation rather than simple transient radical scavenging. As a phenolic bibenzyl compound [55], chrysotoxine can donate hydrogen atoms to lipid peroxyl radicals, forming resonance-stabilized intermediates that terminate chain reactions. Its conjugated aromatic structure may additionally modulate iron-driven redox cycling, collectively contributing to sustained suppression of hemin-induced LDL oxidation and limiting oxLDL formation during early atherogenic processes.

Endothelial activation and subsequent monocyte adhesion are early and critical events in atherogenesis [56,57]. Pro-inflammatory stimuli such as lipopolysaccharide (LPS) activate endothelial cells through toll-like receptor 4–dependent signaling, leading to increased expression of adhesion molecules and enhanced leukocyte recruitment [58,59,60,61]. In the endothelial–monocyte co-culture model employed in this study, LPS markedly increased monocyte adhesion, confirming endothelial activation. Pre-treatment with chrysotoxine significantly reduced monocyte adhesion without affecting endothelial cell viability, indicating a protective effect against inflammatory endothelial responses. Although specific adhesion molecules or intracellular signaling pathways were not directly assessed, the functional monocyte–endothelial adhesion assay was prioritized as an integrated readout of endothelial activation. Nevertheless, the absence of adhesion molecule quantification (e.g., VCAM-1, ICAM-1, E-selectin) limits mechanistic resolution. Future studies should incorporate these markers alongside intracellular oxidative stress endpoints to clarify the upstream pathways modulated by chrysotoxine.

Platelet activation contributes not only to thrombotic complications but also to the progression of atherosclerosis [62]. In this study, chrysotoxine inhibited platelet aggregation induced by arachidonic acid, ADP, and collagen, albeit with differing potencies. ADP-induced aggregation involves signaling through P2Y_1_ and P2Y_12_ receptors, which regulate the initiation and amplification phases of aggregation, respectively [63,64,65]. Chrysotoxine preferentially suppressed the secondary phase of ADP-induced aggregation, suggesting modulation of amplification pathways associated with thromboxane A_2_ generation rather than early calcium-dependent signaling. Collagen-induced aggregation, which depends on phospholipase Cγ2 activation and subsequent thromboxane production for full platelet activation, was delayed and attenuated by chrysotoxine. The most pronounced inhibitory effect was observed against arachidonic acid–induced aggregation, which directly reflects cyclooxygenase-1 (COX-1)–dependent thromboxane A_2_ synthesis [66]. Collectively, these findings suggest that chrysotoxine interferes with platelet activation pathways converging on arachidonic acid metabolism and thromboxane generation.

The relatively higher concentrations of chrysotoxine required to inhibit platelet aggregation likely reflect the experimental use of platelet-rich plasma (PRP), a protein-rich matrix in which hydrophobic small molecules may undergo substantial plasma protein binding, particularly to albumin. Such binding can markedly reduce the free, pharmacologically active fraction of the compound, thereby shifting the effective concentrations required to modulate functional platelet responses into the high-micromolar range compared with those needed to inhibit purified enzymes in cell-free assays [67]. Accordingly, the platelet aggregation data should be interpreted as functional observations that warrant further mechanistic validation.

Cyclooxygenase (COX) enzymes play central roles in inflammation, platelet function, and vascular homeostasis, making them important pharmacological targets in cardiovascular disease [10].While COX-1 inhibition underlies the antiplatelet efficacy of aspirin, selective COX-2 inhibition has been associated with adverse cardiovascular outcomes due to suppression of endothelial prostacyclin production [11,12,13,14,15,16,17]. Molecular docking analyses suggested that chrysotoxine can interact with both COX-1 and COX-2, forming stabilizing interactions with residues involved in substrate access and catalytic activity. These docking results should be interpreted as qualitative support for binding hypotheses rather than as predictors of binding affinity, as docking scores are highly dependent on methodological parameters and simplified representations of protein flexibility and solvation [44].

Consistent with the docking analysis, enzyme activity assays demonstrated that chrysotoxine inhibited both COX isoforms with comparable potency in the low micromolar range. Although these findings indicate non-selective COX inhibition under the experimental conditions used, they do not necessarily predict the extent or balance of COX modulation in vivo. The moderate potency and natural-product scaffold of chrysotoxine may favor partial or context-dependent modulation of COX activity rather than complete enzymatic blockade, which could be advantageous from a cardiovascular safety perspective. In endothelial cells, NF-κB signaling regulates COX-2 expression and broader inflammatory programs [68]. Although NF-κB activation and COX-2 expression were not assessed in the present study, future investigations should determine whether chrysotoxine modulates upstream inflammatory signaling in parallel with its direct effects on COX activity.

Several naturally occurring bibenzyls have been reported to exhibit antioxidant, anti-inflammatory, and antiplatelet activities relevant to the effects observed for chrysotoxine. For example, gigantol and moscatilin have been shown to suppress platelet activation and aggregation in experimental systems [69,70]. In addition, bibenzyls such as moscatilin, gigantol, Aphyllone B, and (−)-Dendroparishiol exhibit antioxidant-associated effects, including radical-scavenging activity and reductions in reactive oxygen species and lipid peroxidation [70,71,72]. Anti-inflammatory effects, including inhibition of LPS-induced mediators and NF-κB–dependent signaling, have also been reported for this chemical class [70,73,74]. Together, these studies support the notion that bibenzyl compounds can concurrently modulate oxidative stress, inflammatory signaling, and platelet function. In this context, the effects of chrysotoxine observed in the present study align with the broader pharmacological profile of related bibenzyls.

Several limitations of this study should be acknowledged. All experiments were conducted in vitro, and the pharmacokinetic properties, bioavailability, and in vivo efficacy of chrysotoxine remain to be determined. LDL oxidation was induced using hemin, and the reaction was terminated with EDTA and BHT. Although this approach effectively limits further oxidative modification, unbound hemin was not removed from the reaction mixture. As no hemin-only control or post-oxidation purification step was included, a contribution of residual hemin to the observed effects cannot be fully excluded. Lipid peroxidation was assessed using the TBARS assay; while additional oxidative stress markers could strengthen mechanistic interpretation, such analyses were not feasible in the isolated LDL model used. Furthermore, endothelial protection was assessed functionally without direct evaluation of adhesion molecule expression or intracellular signaling pathways. Future studies incorporating cellular and in vivo models of atherosclerosis, prostaglandin profiling, and gastrointestinal safety assessments will be required to further define the therapeutic relevance and safety profile of chrysotoxine.

Overall, the present findings demonstrate that chrysotoxine modulates multiple processes relevant to atherogenesis, including oxidative LDL modification, endothelial inflammatory activation, and platelet aggregation. This multi-target profile supports further investigation of chrysotoxine as a plant-derived compound with potential relevance for the prevention or modulation of atherosclerotic disease.

## 5. Conclusions

This study identifies chrysotoxine isolated from *D. pulchellum* as a promising natural antioxidant with notable anti-atherogenic potential. Chrysotoxine demonstrated multifaceted protective effects, including suppression of hemin-induced LDL oxidation, attenuation of LPS-induced monocyte adhesion to endothelial cells, and inhibition of agonist-induced platelet aggregation in vitro.

In addition, in silico molecular docking analyses predicted favorable interactions with cyclooxygenase enzymes, based on optimal binding poses, binding affinity, and key molecular interactions. These computational findings were further supported by cyclooxygenase inhibition assays, providing functional validation of the predicted targets.

Together, these in vitro and computational results strengthen the rationale for further development of chrysotoxine as a protective agent against atherosclerosis. By targeting multiple pathogenic features of the disease, chrysotoxine emerges as a compelling candidate for continued preclinical investigation. These findings warrant further in vivo validation and safety assessment. With additional in vivo validation and comprehensive pharmacological characterization, this plant-derived compound may contribute to the development of new therapeutic strategies to address the complex challenges of atherosclerosis.

## Data Availability

The original contributions presented in this study are included in the article/Supplementary Material. Further inquiries can be directed to the corresponding author.

## Supplementary Materials

The following supporting information can be downloaded at: https://www.mdpi.com/article/10.3390/biom16030379/s1, Figure S1. Effect of Chrysotoxine on relative electrophoretic mobility (REM) in hemin-induced LDL oxidation at various times of incubation corresponding to Figure 3. Figure S2. Summary of the human cell lines used in this study corresponding to Figure 4 and Figure 5. Figure S3. Effects of chrysotoxine on agonist-induced platelet aggregation corresponding to Figure 6. Figure S4. Docking overlay of chrysotoxine with COX-1 corresponding to Figure 7. Figure S5. Docking overlay of chrysotoxine with COX-2 corresponding to Figure 7. Figure S6. Effects of chrysotoxine on COX-1 and COX-2 enzyme activities corresponding to Figure 8. Table S1. Detailed information on the chemicals and reagents used in this study. Table S2. Molecular docking summary for 2OYE (Cyclooxygenase-1). Table S3. Molecular docking summary for 4COX (Cyclooxygenase-2).
