# Supplementary material for: Chrysotoxine Attenuates Key Atherogenic Processes via Antioxidant, Anti-Inflammatory, and COX-Dependent Antiplatelet Mechanisms
Source: Biomolecules. 2026 Mar 3;16(3):379. doi: 10.3390/biom16030379 (PMC13024289; doi:10.3390/biom16030379)
Supplement: Supplementary file 1 [file biomolecules-4134253-supplementary.pdf › biomolecules-4134253-supplementary.pdf]

Article

Chrysotoxine Attenuates Key Atherogenic Processes via Anti-oxidant, Anti-Inflammatory, and COX-Dependent Antiplatelet Mechanisms

Fozia Rustamani[1], Hla Nu Swe[1], Su Wutyi Thant[1], Jeeradej Moonrut[2], Boonchoo Sritularak[3,4], Ponlapat Rojnuckarin[5], Nonthaneth Nalinratana[4,6] and Rataya Luechapudiporn[4,6,7,*]

[1] Pharmaceutical Sciences and Technology Program, Faculty of Pharmaceutical Sciences, Chulalongkorn University, Bangkok, Thailand

[2] Doctor of Philosophy Program in Pharmacology, Faculty of Graduate School, Chulalongkorn University, Bangkok, Thailand

[3] Department of Pharmacognosy and Pharmaceutical Botany, Faculty of Pharmaceutical Sciences, Chulalongkorn University, Bangkok, Thailand

[4] Center of Excellence in Natural Products for Ageing and Chronic Diseases, Faculty of Pharmaceutical Sciences, Chulalongkorn University, Bangkok, Thailand

[5] Department of Medicine, Faculty of Medicine, Chulalongkorn University, Bangkok, Thailand

[6] Department of Pharmacology and Physiology, Faculty of Pharmaceutical Sciences, Chulalongkorn University, Bangkok, Thailand

[7] Faculty of Pharmaceutical Sciences, Kasetsart University, Bangkok, Thailand

* Correspondence: Department of Pharmacology and Physiology, Faculty of Pharmaceutical Sciences, Chulalongkorn University, Bangkok 10330 Thailand: E-mail address: rataya.l@pharm.chula.ac.th

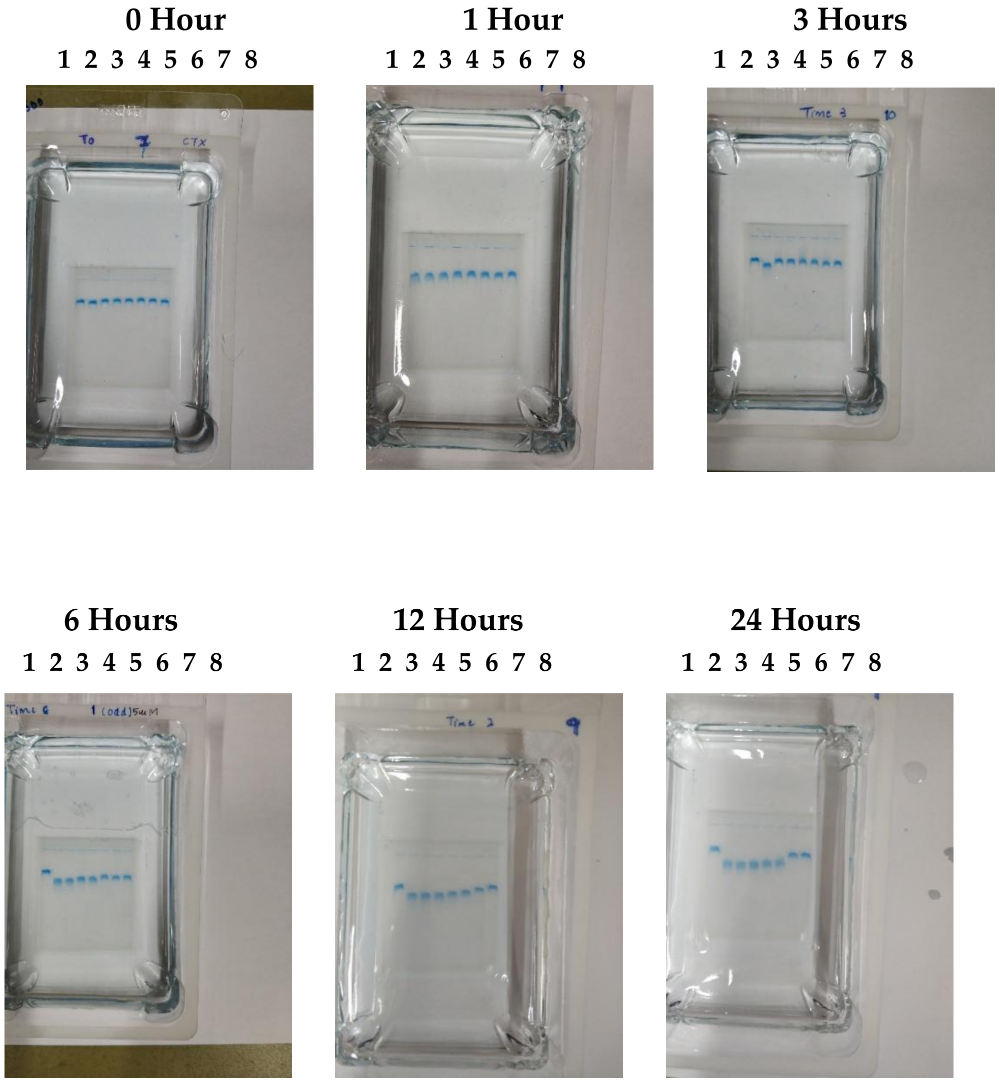

Supplementary Figure S1. Effect of Chrysotoxine on relative electrophoretic mobility (REM) in hemin-induced LDL oxidation at various times of incubation. Representative REM in agarose electrophoresis. Lane 1: nLDL, lane 2: he-oxLDL, lane 3: Trolox 0.5 μM, lane 4: Trolox 1 μM, lane 5: CTX 0.5 μM, lane 6: CTX 1 μM, lane 7: CTX 2 μM, lane 8: CTX 4 μM.

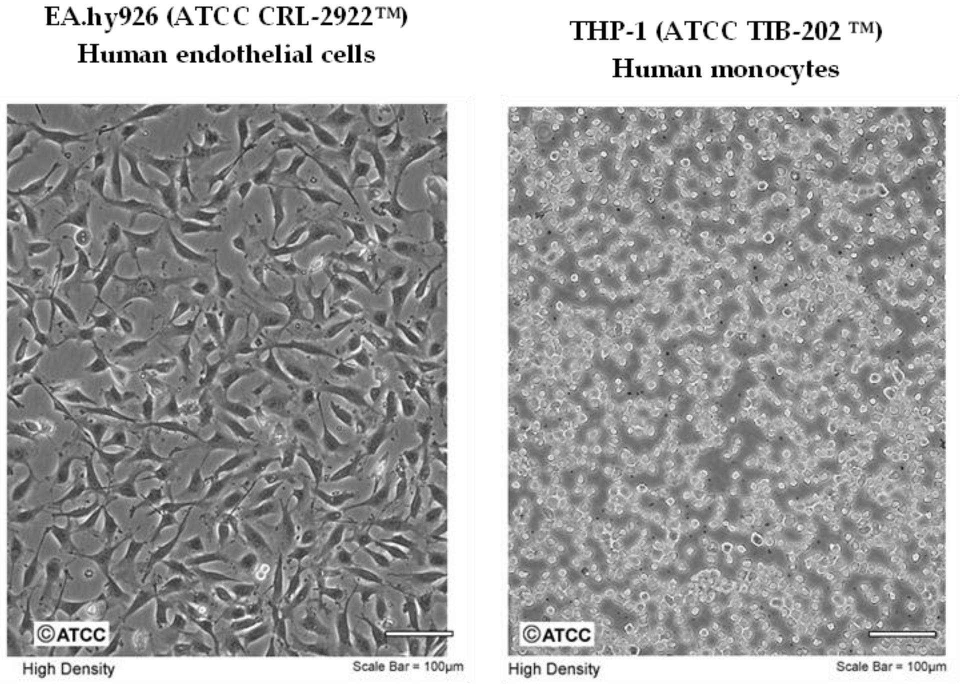

Supplementary Figure S2. Summary of the human cell lines used in this study.
The endothelial cell line EA.hy926 (ATCC CRL-2922™) and the monocytic cell line THP-1 (ATCC TIB-202™) were sourced from the American Type Culture Collection (ATCC, USA) and maintained according to standard culture conditions.

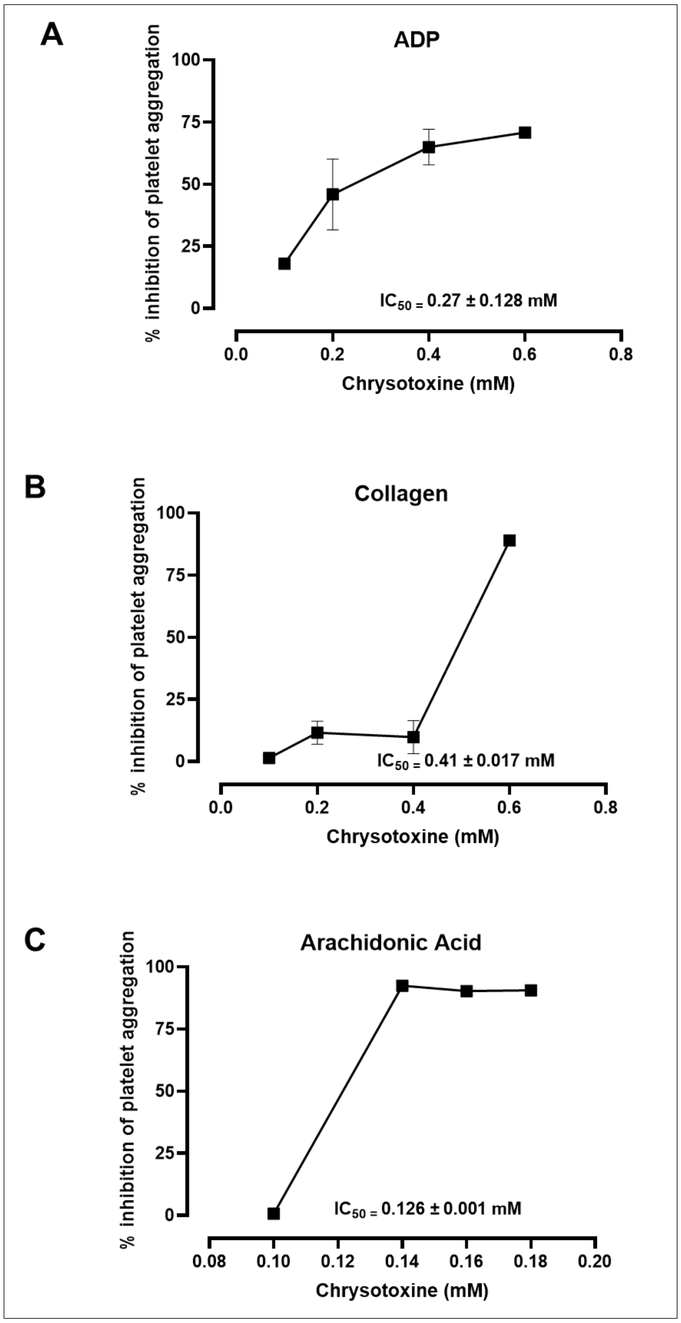

Supplementary Figure S3. Effects of chrysotoxine on agonist-induced platelet aggregation. Chrysotoxine inhibited platelet aggregation in a concentration-dependent manner when induced by (A) adenosine diphosphate (ADP, 4 μM), (B) collagen (2 μg/mL), and (C) arachidonic acid (AA, 0.5 mM). Results are expressed as the percentage inhibition of platelet aggregation and are presented as mean ± SEM (n = 3).

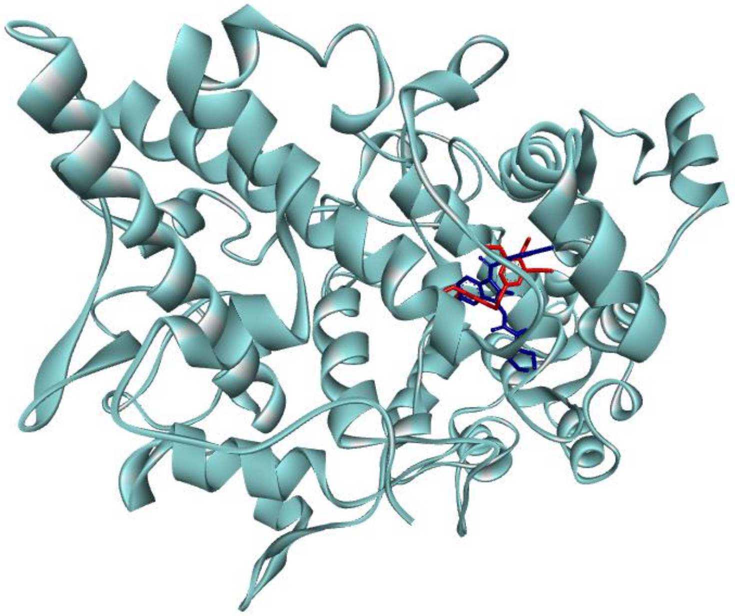

Supplementary Figure S4. Docking overlay of chrysotoxine with COX-1.
The native reference ligand (IM8) is represented in blue, whereas chrysotoxine is shown in red within the COX-1 active site, highlighting their spatial orientation and binding overlap.

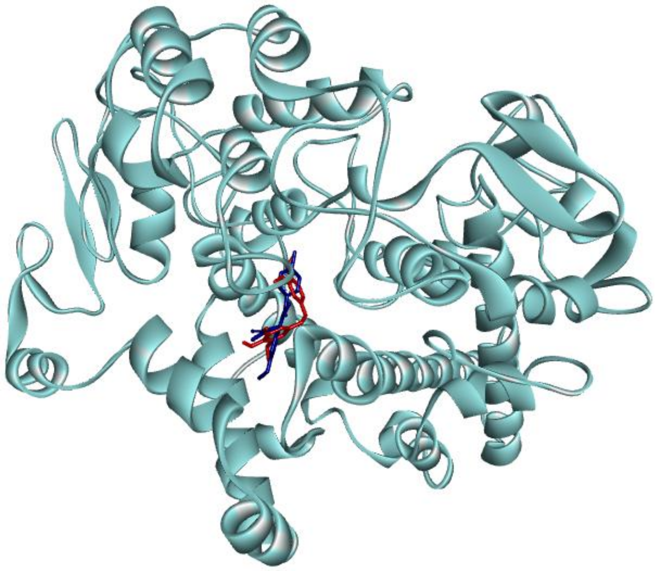

Supplementary Figure S5. Docking overlay of chrysotoxine with COX-2.
The native reference ligand (IMN) is shown in blue, while chrysotoxine is depicted in red within the COX-2 binding pocket, illustrating the alignment and potential interaction sites of both molecules.

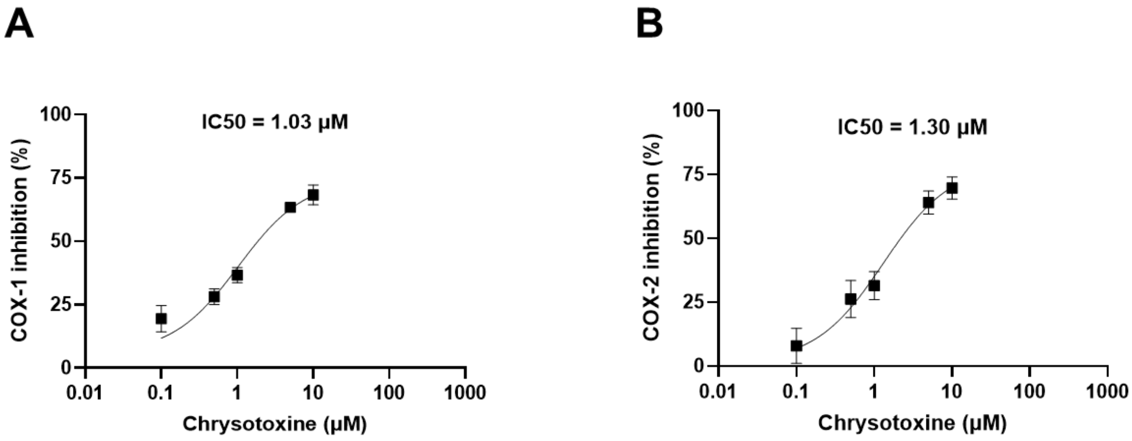

Supplementary Figure S6. Effects of chrysotoxine on COX-1 and COX-2 enzyme activities. Chrysotoxine exhibited concentration-dependent inhibition of COX-1 and COX-2 enzyme activities, with IC_{50} values determined as 1.03 μM and 1.30 μM, respectively. Data are presented as percent inhibition of COX activity (means ± SEMs; *n* = 3).

Supplementary Table S1. Detailed information on the chemicals/reagents used for the research study.

Chemicals/ Reagents	Cat. No.	Manufacturer
Trisodium citrate dihydrate	S4641	Sigma-Aldrich, Merck Group
adenosine 5′-diphosphate (ADP)	01897	Sigma-Aldrich, MilliporeSigma
Arachidonic acid >95.0% (GC)	SIA-10931-250MG	Sigma-Aldrich, MilliporeSigma
Collagen	HLUS-5368	HELENA Laboratories
acetylsalicylic acid (ASA)	50-78-2	Sigma-Aldrich, MilliporeSigma
Dimethyl Sulfoxide> 99.0% (GC)	TCI-D0798-500G	Tokyo Chemical Industry Co., Ltd.
Ethylenediaminetetraacetic acid disodium dehydrate	E1644	Sigma-Aldrich, MilliporeSigma
Potassium bromide	P0838	Sigma-Aldrich, MilliporeSigma
Potassium chloride	P9541	Sigma-Aldrich (Merck Millipore)
Disodium phosphate	M8266 (dibasic)	Sigma-Aldrich (Merck Millipore)
Potassium dihydrogen phosphate	P0662	Sigma-Aldrich, MilliporeSigma
Butylated hydroxytoluene (BHT)	W218405	Sigma-Aldrich, MilliporeSigma
Sodium dodecyl sulfate (SDS)	L4509	Sigma-Aldrich (Thermo Fisher)
Trichloroacetic acid (TCA)	T6399	Sigma-Aldrich, MilliporeSigma
Thiobarbituric acid	T5500	Sigma-Aldrich, MilliporeSigma
Sodium chloride (NaCl)	31434	Merck (Thermo Fisher)
Agarose powder	161-3101	Bio-Rad Laboratories
Coomassie blue solution	161-0400	Bio-Rad Laboratories
Tris-acetate-EDTA (TAE) buffer	161-0733	Bio-Rad Laboratories
D-MEM	1TFS-1CC-12800017	Gibco™, Thermo Fisher Scientific
RPMI 1640 Medium	11875093	Gibco™, Thermo Fisher Scientific
Fetal Bovine Serum	1TFS-1RS-A5256701	Gibco™, Thermo Fisher Scientific
Penicillin-Streptomycin	1TFS-1CC-15140122	Gibco™, Thermo Fisher Scientific
Trypsin-EDTA	1 TFS-1CC-25200056	Gibco™, Thermo Fisher Scientific
Thiazolyl Blue Tetrazolium Bromide 98%	SIA-M2128-500MG	Sigma-Aldrich, MilliporeSigma
Phosphate buffered saline powder, pH 7.4	SIA-P3813-10PAK	Sigma-Aldrich, MilliporeSigma
Calcein, AM	3N-C3099	Invitrogen™, Thermo Fisher Scientific
COX Fluorescent Inhibitor Screening Assay Kit	CAY-700100	Cayman Chemical
Lipopolysaccharides from Escherichia coli O111:B4 purified by phenol extraction	SIA-L2630-10MG	Sigma-Aldrich, MilliporeSigma

Supplementary Table S2. Molecular Docking Summary – 2OYE (Cyclooxygenase-1)

**Parameter**	**Details**	
**Complete Protein Name**	INDOMETHACIN-(R)-ALPHA-ETHYL-ETHANOLAMIDE BOUND TO CYCLOOXYGENASE-1	
**Protein PDB Code**	**2OYE**	
**Native Ligand (IM8)**	*2-[1-(4-CHLOROBENZOYL)-5-METHOXY-2-METHYL-1H-INDOL-3-YL]-N-[(1R)-1-(HYDROXYMETHYL)PROPYL]ACETAMIDE*	
**Positive Control**	Arachidonic Acid (AA)	
**Experimental Ligand**	Chrysotoxine (Phenol, 4-[2-(3,4-dimethoxyphenyl)ethyl]-2,6-dimethoxy-4-[2-(3,4-dimethoxyphenyl)ethyl]-2,6-dimethoxyphenol4-(3,4-dimethoxyphenethyl)-2,6-dimethoxyphenol)	
**RMSD (Redocked vs. Native Pose)**	**0.657 Å**	
**Ligand**	**Role**	**Binding Affinity (kcal/mol)**
IM8 (Native Ligand)	Reference	**-8.2**
Arachidonic Acid	Positive Control	**-7.8**
Chrysotoxine	Experimental Ligand	**-8.1**

Supplementary Table S3. Molecular Docking Summary – 4COX (Cyclooxygenase-2)

**Parameter**	**Details**	
**Complete Protein Name**	CYCLOOXYGENASE-2 (PROSTAGLANDIN SYNTHASE-2) COMPLEXED WITH A NON-SELECTIVE INHIBITOR, INDOMETHACIN	
**Protein PDB Code**	4COX	
**Native Ligand (IMN)**	*Indomethacin (2-[1-(4-chlorophenyl)carbonyl-5-methoxy-2-methyl-indol-3-yl]ethanoic acid)*	
**Positive Control**	Arachidonic Acid (AA)	
**Experimental Ligand**	Chrysotoxine (Phenol, 4-[2-(3,4-dimethoxyphenyl)ethyl]-2,6-dimethoxy-4-[2-(3,4-dimethoxyphenyl)ethyl]-2,6-dimethoxyphenol4-(3,4-dimethoxyphenethyl)-2,6-dimethoxyphenol)	
**RMSD (Redocked vs. Native Pose)**	**0.457 Å**	
**Ligand**	**Role**	**Binding Affinity (kcal/mol)**
IMN (Native Ligand)	Reference	**-9.2**
Arachidonic Acid	Positive Control	**-7.7**
Chrysotoxine	Experimental Ligand	**-7.1**
